# Supplementary material for: Care for critically and terminally ill patients and moral distress of physicians and nurses in tertiary hospitals in South Korea: A qualitative study
Source: PLoS One. 2021 Dec 16;16(12):e0260343. doi: 10.1371/journal.pone.0260343 (PMC8675648; doi:10.1371/journal.pone.0260343)
Supplement: Attachment — Submitted filename: Response to Reviewers.docx [file pone.0260343.s001.docx]

**Response to Review**

**Reviewer #1**

| **Comments** | **Response** |
| --- | --- |
| Thank you for the opportunity to review this manuscript on the increasingly important topic of moral distress among clinicians.  The authors have presented the data well and it is well written especially as English is not their first language. Additionally, it is important to have data from different countries as the embedding of palliative care into clinical care differs in many settings.  The authors have highlighted important cultural differences in particular in both clinician and family views of the role of palliative care. It is disappointing that it is seen as "giving up" and that "nothing more can be done". This indicates the need for education both at a clinical and community level which the authors highlight in the Discussion. | We appreciate your review and thorough feedback. Your comments helped us to improve the manuscript. |
| The themes are well described however, the findings needed to be structured more around the Semi-structured interview questions. The responses to these questions do not appear in the manuscript at all. The paper is more focussed on the failings of providing end of life care rather than the moral distress that this causes. | Drawing upon the reviewers’ comments, we have reorganized the manuscript to focus more clearly on moral distress, which was aligned with the interview questions. |
| I would also like to see a definition of critical incident techniques which was the theoretical basis of the analysis. | Drawing upon three reviewers’ comments regarding the hybrid methodology, we have deleted the section on the critical incident technique and focused on grounded theory. |
| The Abstract needs to be stronger to reflect the findings and the conclusion needs to be more aligned with the question of moral distress - which is the theme of the study - hence the statement from the manuscript that "Medical practitioners experienced moral distress either when providing futile life-sustaining treatment or failing to provide comfort care”. This I see as the key message rather that a statement that the study demonstrated that end-of-life care is neglected in acute care settings or a listing of the ways end of life care has failed.  The study brings important insights, however it needs to be more focussed on the main question of the study. | Thank you for your suggestions. We have reorganized the results to give prominence to the moral distress: to what participants wanted to do for their patients, to what hampered them, and to what consequences of moral distress. The statement that you saw as the key message has been clarified in the Discussion section of the revised version. |

**Reviewer #2**

| **Comments** | **Response** |
| --- | --- |
| - Thank you for the opportunity to review your manuscript, ‘Imbalanced end-of-life care and moral distress in tertiary hospitals: A qualitative study’. The topic is a worthy one and the study shines a light on the experiences of physicians and nurses in Korea in end of life care that causes them moral distress. However, the manuscript requires attention in the following areas before it is ready for publication. | We appreciate your suggestions. Your comments helped us to improve the manuscript. |
| 1. Title: should include participants, as it is not clear whose moral distress was examined. | Thank you for your suggestion. We have changed the manuscript’s title to “Care for critically and terminally ill patients and moral distress of physicians and nurses in tertiary hospitals in South Korea: A qualitative study.” |
| 2. Abstract:  a. Suggest you do not use the term ‘medical practitioners’ to describe the physician and nurse participants, as this term signifies physicians in many parts of the world. Suggest the terms ‘health professionals’ or ‘clinicians’ would be more universally correct. | We have changed the term to healthcare professionals, which signifies both doctors and nurses. |
| b. The assertion that the study demonstrates that end of life care is neglected in Korean hospitals is too strong/too great a leap for a qualitative study of the perspectives of physicians and nurses. Please revise this statement so that it is more in keeping with the methods/objectives of your investigation. | Drawing upon the reviewers’ comments, we have reorganized the manuscript to focus it on moral distress and have avoided making an excessively strong argument in the revised version. |
| 3. Introduction:  a. Please explicitly define end of life care, as this is your key concept, and it can be interpreted differently. For example, in Australia end of life care is defined as that which aims to optimise quality of life for persons likely to die in the next 12 months by addressing suffering, promoting dignity and function, respecting their needs and preferences, and supporting their family, including into bereavement. Is this what you are referring to by end of life care? | Thank you for addressing this point. The population of patients addressed in the interviews was not restricted to those whose life expectancy was less than six or twelve months. For example, some interviews talked about stroke patients and babies with anencephaly, who do not fit the definition of end-of-life care patient. Thus, we changed the term *end-of-life care* to *care for critically and terminally ill patient*s in the revised version to encompass the wide range of care and moral distress that occurred in the tertiary hospitals. |
| b. Suggest you revise use of the term ‘a good death’ (page 3), as this concept differs from end of life care, and furthermore it is a loaded and variously interpreted term.  c. Suggest use of less euphemistic language than ‘meet their demise there’ (paragraph 2, page 3) e.g. ‘die there’. | Thank you for your suggestion. We have deleted the euphemistic language in the revised version. |
| d. Please provide a reference/s to substantiate the low level of satisfaction with end of life care in particular regions of the world (page 3). | We have deleted this part. |
| e. The introduction should include information about the end of life care needs, services and health care policy in Korea, to communicate to the reader the context in which the study was conducted. | We appreciate your suggestion. We have included in the introduction to the revised version the palliative care services provided in tertiary hospitals, the supplies of hospices, and the health care policy in Korea. |
| f. The last paragraph of the introduction (the aim of the study) contains two different and seemingly dissonant objectives. The first was to examine physicians’ and nurses’ subjective experiences of moral distress related to end of life care. The second was to identify the organisation and systemic barriers to EOL in the hospitals, which strikes me as being more suited to an objective assessment, and too great a leap from the first objective. Please re-consider the scope of examination of the topic that was possible using qualitative methods. | This suggestion is related to the main point raised by three reviewers. Drawing upon their comments, we have reorganized the manuscript along with the first objective. We agree that the original made an unsupported leap from subjective experiences of moral distress to systemic barriers to end-of-life care. The revised version is structured according to what the participants pursued, what institutional constraints hampered them, and what consequences resulted from the moral distress. |
| 4. Methods:  a. The manuscript states that the COREQ reporting guideline informed the study methods. This statement is problematic for two reasons. Firstly, reporting guidelines are to guide study reporting, not study methods. Secondly, the study has not been reported according to the COREQ, as several items are missing. Rather than pointing each of these out individually, my suggestion is that you ensure that all items of the COREQ are reported in your revised manuscript. | Thank you for your suggestion. We have ensured that all the items of the COREQ are reported in the revised manuscript. |
| b. Table 1 belongs in the results section, not the methods, as does the information about duration of the interviews. | We have moved Table 1 to the Results. |
| c. Critical incident technique has a particular analytic method, whereas you used a grounded theory analysis approach. Currently, the reporting suggests an unsound hybrid qualitative approach, especially as the findings are not reported in line with critical incident technique. Please revise this section to justify why you used the hybrid approach. | Drawing upon three reviewers’ comments regarding the hybrid methodology, we have deleted the section on the critical incident technique and focused on grounded theory. |
| d. Analysis: last line - what specific type of bias did you minimise? Also, because you have not reported your positions, backgrounds and reflexivity process (which the COREQ calls for), it is difficult for the reader to be convinced that you fully addressed the minimisation of researcher bias. | The revised version clarifies this point. We have added, “Through multiple rounds, all the members from diverse positions (medical student, doctoral candidate, and professor) and various backgrounds (medical anthropology, medicine history, nursing, palliative care, and oncology) examined the interview data in a reflexive manner, and this process contributed to minimizing a biased interpretation.” |
| 5. Findings:  a. An introductory paragraph, including the characteristics of participants and interview, and summary of the themes and sub-themes, is required. Also, I was left wondering what proportion of the sample experienced moral distress. | We have added an introductory paragraph: “Among the 22 participants, 21 reported that they experienced moral distress when they knew what was right for the patients but failed to pursue their values and beliefs due to institutional constraints. One nurse working in the intensive care unit addressed the emotional distress caused by caring for the dying patients rather than the moral distress. The data analysis identified what the participants valued for the patients (Table 3).” |
| b. In reporting the findings, it is important not to over-generalise. For example, the statement that procedures and treatments were not provided in patients’ best interests (page 8) should be modified, as you are recounting health professionals’ subjective perspectives only. | Yes, we have tried not to over-generalize the findings. The statement regarding the patients’ best interest reflected what the participants thought, and we have clarified this point in the revised version. |
| c. The statement “Because obtaining a DNR order means cessation of treatment…” requires revision, as a DNR order means no cardiopulmonary resuscitation, not no treatment overall. | We absolutely agree with you. Unfortunately, however, a DNR order was regarded by the participants in tertiary hospitals as giving up on the patient. We have made this point clearer in the revised version. |
| d. I believe that the lack of exemplar quotes in the reporting of the ‘Main contributing factors’ theme adversely affects the strength and trustworthiness of your interpretations. While they are available in the supplementary file, their absence in the narrative means that the findings are reported in a disjointed way. Please consider formatting the findings in a consistent way throughout, and provide exemplar quotes for each sub-theme. | Thank you for your suggestion. We have included exemplary quotes for each subtheme in this revised version. |
| 6. Discussion:  a. The first paragraph of the discussion is clear and reflects the findings.  b. However, at times you introduced new findings in the discussion that were not earlier reported. For example, I can’t see anything that refers to patients’ pain in the findings, but it is referred to on page 13. Similarly, there were no data on participants wanting to leave their work, yet it is referred to on page 14. Only items reported in the findings should be discussed. | Thank you for your suggestion. We have revised the main results so as to discuss only the items reported in the findings. |
| c. As per my earlier comments about there being too great a leap from investigating participants’ moral distress to interpretations about systems/macro level change, I suggest you rein in recommendations about macro-level transformation; or at least, provide more specific recommendations about required practice and system change. | We appreciate your comments. We have revised the results and discussion to provide well-integrated suggestions. |
| d. Please provide a conclusion. | The revised version includes a conclusion. |
| 7. References: A key missing reference is: ‘Re-defining moral distress: A systematic review and critical re-appraisal of the argument-based bioethics literature’ https://journals.sagepub.com/doi/abs/10.1177/1477750919886088 | This reference contributed to the revised manuscript. Thank you very much. |
| 8. Figure: I found it strange that moral distress of health professionals was the end point, whereas really it was your starting point. Also, it suggested to me a focus on health professionals’ well-being, rather than patients or their families, who are the primary focus of end of life care. I suggest that some more thinking is required about how to best convey the findings of your study diagrammatically. | In restructuring and refocusing the Results and Discussion sections, we deleted Figure 1. |

**Reviewer #3**

| **Comments** | **Response** |
| --- | --- |
| Thank you for the opportunity review this paper, which explores end of life care and moral distress in tertiary hospitals in Korea. The authors have interesting data and raise important points, however, overall there are a number of methodological issues with the paper and overall, I find it quite confusing.  I think that this paper needs a bit of re-focussing and I wonder if authors are trying to do too much by exploring moral distress and imbalance of services. Regarding the latter, this feels a little bit tagged on and I wonder whether this would be a stronger paper I it focussed upon causes of moral distress in S Korean acute settings – using organization of services aspects as discussion.  As currently written, I think the introduction needs to more clearly contextualise the paper in terms of: known causes of moral distress in the context of palliative care, and description of the South Korean health system – it is only in the results that palliative and hospice services are referred to. Methods also need improved description and justification of approach.  This is interesting work in need of improvement ahead of being publishable work – I have commented in more detail on specific sections below. | We appreciate your suggestions, which helped us to improve the manuscript.  Drawing upon three reviewers’ suggestions, we refocused on what the physicians and nurses wanted to pursue, what institutional constraints hampered them, and the consequences of the moral distress. We have also included background information on such matters as the available palliative care services, the supplies of hospices, and health care policy in South Korea. |
| Introduction  The concept of moral distress is interesting, but it would be useful to have some examples here – what would be examples of the ‘diverse and even contradictory ethical and cultural values, as well as legal restrictions’ in which clinicians works in S Korea?  The claim that “tertiary hospitals serve as the starting points of end-of-life care” requires justification. You must forgive my lack of contextual knowledge of the S Korea healthcare system, but it would be useful to have some description of what other services/alternatives are available to the acute setting i.e. we learn that hospice is available in the results, is the suggestion that some people go to acute care when they more appropriately be under hospice care? Also, examples are given of western countries – many of which will have hospital palliative care teams (even if satisfaction remains low) – are any such services available in S Korea?  Overall, I don’t feel that the introduction justifies the aims. It feels more like you are focussing upon factors which contribute to moral distress in acute hospitals? The additional aim regarding organization of care feels a little tagged on and without improved description of the healthcare system overall, difficult to understand. | We have added examples of the ethical values and legal restrictions associated with caring for critically and terminally ill patients, and we have added background information on South Korea’s health care system. |
| Methods  Most importantly, I am not satisfied that the authors use Grounded Theory, which is not something which can simply be applied as a mode of analysis. GT applies far earlier than the analysis stage and would also require continuing analysis, used to inform further data collection. The analysis process which the authors describe sounds entirely suitable and of due rigor, but is not GT and requires different description. | We absolutely agree that grounded theory cannot simply be applied as a mode of analysis. The characteristics that you mentioned in regard to grounded theory reflected what we tried to accomplish in this research. To make this point clearer, we have added, “The categories and themes addressed in the previous interviews were redefined in subsequent interviews and informed further data collection.” Although grounded theory, as a methodology and not merely a method, encompasses the research design stage and theorization, we describe how we employed grounded theory in a more or less limited way because examining grounded theory was not the aim of this research. |
| As for the sampling strategy, authors state that ‘purposive maximum variation’ sampling, but also that participants were recruited through open advert. Please make clear how this approach allowed a diverse range of views and also provide comment on how the sample size was agreed and its representation of the workforce within acute hospitals across departments. | When no new issues were raised by one department (e.g., the Emergency Intensive Care Unit) or one position (e.g., nurses), we determined that we had reached theoretical saturation and closed recruitment. Due to limited time and resources, the sample size was small in some departments (e.g., Family Medicine) and positions (e.g., fellows). Further study is needed to examine cross-departmental differences. |
| Thank you for including the interview questions – I think they look good but also echo that organization of care was not a key aim. | We did not intend to limit the conflicts to those between individual and institutional constraints, but all except one of the participants described organizational constraints as their concern. Because we drew upon grounded theory, the interview questions had evolved centering on the institutional constraints. |
| Results  First line states that ‘overtreatment and a lack of care emerged from the interviews as issues causing the participants’ moral distress’. I feel that it needs to be established that participants did experience moral distress before making this claim. Did participants identify as having moral distress in interviews? Was this part of an inclusion/exclusion criteria? It is noted as a limitation that ‘all participants had ‘moral distress’ though I am not satisfied that this has been established – the recruitment poster only refers to inviting people who had provided terminal care. | We appreciate your questions. The recruitment poster invited people who had taken care of terminally and critically ill patients and had experienced any moral difficulties. We did not limit recruitment by the criteria of “care for dying patients” or “moral distress” because we aimed to examine the issues that physicians and nurses would address in subjective ways. Before the pediatric staff were enrolled, for example, we had no idea that the physicians and nurses counted the care burden of a bedridden patient’s family. We believe that this aspect reveals the complicated role of tertiary hospitals in dealing with patients at the threshold of life and death and highlights the medical advances that have created a “gray zone” between life and death. Also, we did not use the experience of moral distress as an inclusion/exclusion criterion, but 21 of the 22 participants expressed that they had experienced conflicts between what they wanted to do for the patient and what was allowed by organizational constraints. We have clarified this point in the revised version. |
| Findings on Slow Code are heartbreaking, but require setting up better in the introduction to achieve full impact. In what circumstances do legal issues arise here to cause ‘fear of lawsuits?’ RE: DNRs, please clarify what is meant by ‘cessation of treatment’ – I would expect that people would continue to receive usual care in this circumstance, but would not be resuscitated. | We have included in the Introduction an explanation of the legal issues and the meaning of obtaining a DNR. |
| In ‘Lack of end-of-life care resources,’ it’s stated that palliative and hospice care are marginalised in the hospital system, but this is the first suggestion that these services may be available at all! Please clarify.  Euthanasia is mentioned within ‘main results’ but this is the first use of the word in the manuscript. Is euthanasia practiced in S Korea? | We have described in the Introduction the palliative services provided in tertiary hospitals. Regarding euthanasia, we tried to address the fact that moral distress in end-of-life care has tended to focus on life-sustaining treatment and that other issues have tended to be neglected. We have clarified this point in the revised version. (Euthanasia is not practiced in Korea.) |
| I feel that more discussion is needed regarding the patient profile within an acute hospital in S Korea. Continuing life sustaining treatment can be a barrier to appropriate care, but all kinds of patients will be present in an acute setting, many of whom for which life sustaining therapy would be appropriate. Please comment on this issue and the prevalence of people with terminal illness in this setting. | Thank you for your suggestion. We have more clearly explained that the participants regarded life-sustaining treatment as meaningless only when the patient seemed to be at the terminal stage. Also, we have added cases in which the participants wanted to continue the treatment but could not do so. |
| Discussion  The Discussion as currently written is quite vague and a little confusing. E.g. the sentence “if a doctor does not believe that he or she will be supported by the hospital system when a patient’s relatives file a lawsuit, it is hard for the doctor to pursue the patient’s best interests or wishes.” Is somewhat alarming and poorly explained.  I would suggest that this section needs re-writing to both review the causes of moral distress and make clear its consequences. Description included within this section re: available services in acute settings, should come much earlier (introduction) so that Discussion can follow here.  “The six contributing factors that participants addressed are consistent with the themes and root causes reported in previous studies” – what are the contributing factors and what do they contribute to? – please refer to these factors or reference the figure earlier. | In line with the revisions made in the Introduction, Methods, and Results, we have revised the Discussion to clarify our arguments. |
