# Supplementary material for: Care for critically and terminally ill patients and moral distress of physicians and nurses in tertiary hospitals in South Korea: A qualitative study
Source: PLoS One. 2021 Dec 16;16(12):e0260343. doi: 10.1371/journal.pone.0260343 (PMC8675648; doi:10.1371/journal.pone.0260343)
Supplement: Attachment — Submitted filename: Response to Reviewers1.docx [file pone.0260343.s002.docx]

**Response to Review**

**Reviewer #2**

| **Comments** | **Response** |
| --- | --- |
| Reviewer #2: Thank you for the opportunity to comment on your revised manuscript, 'Care for critically and terminally ill patients and moral distress of physicians and nurses in tertiary hospitals in South Korea: A qualitative study'.  The revised version is much more coherent and well integrated, and you have addressed most of the reviewers' comments well.  However, there are a few important areas still needing attention before the manuscript is ready for publication, as follows:  Title/abstract/aim: The phrasing of what you examined is imprecise in these parts of the report. Moral distress is experienced by persons in specific situations, so a clearer and simpler way of expressing what you examined is exactly as you expressed it in the study design section i.e. ''...'health professionals' experience of moral distress related to caring for critically and terminally ill patients'. My advice is that you use this better phrasing at all times throughout the study report. | We appreciate your review and thorough feedback. Your comments truly helped us to improve the manuscript. |
| Introduction: Bravo - this is much more informative and focused. However, I suggest a final refinement of the first paragraph by rounding up the percentages to whole numbers and tying in similar findings e.g. 49.9% of hospital death in the UK and 50% of hospital deaths in Australia is basically the same proportion and could be expressed as 'Half of people in the UK and Australia die in hospital compared to more than three-quarters (76%) of people in Korea (and btw, is that figure for South Korea only or all of Korea?). Also, given much of the previous research related to your topic was likely conducted in the US, what are the US stats? | Drawing upon the reviewer’ comments, we have added the U.S. stats and made a comparison between the East Asian countries and North-Euro American countries to reveal that this hospital death becomes more salient in the East Asia. |
| Aim statement (pages 4-5): is the first statement your aim and the second statement your objectives? As presently this reads as two somewhat different aims. Suggest revise this section for improved clarity. | Thank you for your suggestion. We have revised this section. |
| Table 1: The red text indicates you revised this information, yet the wording appears exactly the same as the original version. Please would you explain this point? (especially as interview questions can't be changed retrospectively) | The interview questions in the first manuscripts were:   1. Have you experienced ethical concerns or moral conflicts while providing end-of-life care? 2. Did you feel any physical or psychological burden because of these moral conflicts? 3. Did these moral conflicts affect the care that you provided? 4. How did you try to solve the problem? 5. What advice would you like to give someone who is experiencing a similar problem? 6. What is your suggestion for preventing and/or solving this problem?   Whereas, in the previous revised manuscripts, we had presented the interview questions as:   1. Have you experienced ethical concerns or moral conflicts while caring for critically and terminally ill patient?    1-1. What did you want to do for the patient?    1-2. Why did your ethical concerns or moral conflicts occur? 2. Did you feel any physical or psychological burden because of these moral conflicts? 3. How did you try to solve the problem? 4. What advice would you like to give someone who is experiencing a similar problem?   We had revised the term ‘end-of-life care.’ Also, we have added the sub-questions under the first one. This does not mean that the interview questions are changed retrospectively, but that they were the original follow-up questions. We have added them to make clear the research purpose. |
| Analytic approach: I share Reviewer 3's concern that you have not fully applied grounded theory. I can see that you have used elements of grounded theory, so my recommendation to address this issue is that you state you used a qualitative approach based on grounded theory, and then state exactly what the applied aspects were. It is also important to state the rationale for your choice of analytical method. Given there were existing data on moral distress in health professionals in relation to care of people likely to die, a grounded theory approach seems inappropriate, unless you choose to use it because there were little existing data on this topic in South Korea. | There is very little data on moral distress that healthcare professionals in acute care settings in East Asia, not to mention in South Korea, experience while caring for critically-ill and terminally-ill patients. We have added: “We adopted the grounded theory because this is a useful analytic tool when a research purpose is to uncover the problems faced by participants within a particular context.” Also, we have made clear that we had created core categories, key themes with memos while coding. |
| Findings: - This section is now more focused and clear, but there are many author statements made without the addition of supporting quotes. For your study findings to be trustworthy, every assertion you make about what participants said should be backed up by an exemplar quote. By doing this, you are also allowing the reader to hear the participants' voices, which provides a more compelling narrative. | We have added more quotes to make clear that we had drawn upon the participants’ voices and to support our findings. |
| - The quote from the paediatric physician indicated to me that he/she was tempted to or in fact had dissembled (or more bluntly, been dishonest) with others in the team or even the family of the critically ill child about their clinical interventions. It also suggests (although this may not be what they meant) that they took an active role in hastening the death of the child, which is euthanasia, unethical and unlawful. However, the participant may simply have meant that they allowed (or wished to allow) that the child die a natural death through the removal of life sustaining treatment, which is ethical and lawful IF done transparently, with supportive care, and with the consent of those who are the primary decision makers for the child, which is almost always their parents/family. Given that you understand the context in which this quote sits, and the importance of distinguishing clinicians' intentions in their care of people at the end of life, please provide more explanation of what the participant meant, and explicitly acknowledge its implications in the discussion. For example, if you discerned that participants were not always honest/transparent in their clinical communication and actions, what was driving this troubling issue? I realise that addressing findings that do not align with ethical practice is challenging, but am sure that you will be able to do this well in a way that maintains respect and compassion for the participants while honoring what is owed to patients and their families. | We have realized that readers could misunderstand what the participant meant. The participant did not mean that he actually had done something to hasten his patient’s death. In order to make clear this point, we have added another quote from another pediatric physician. The quote in question was an expression of scepticism that he felt. When seeing that his practice produced a bedridden child and an exhausted mother, he was not sure if he did the right thing. We believe that the revised manuscript has clarified what he meant and what we meant. |
| - Similarly, in the quote from the nurse about pain relief, what exactly did he/she mean? Did he/she mean that the pain relief should be individualised to the needs of the person? As it is entirely reasonable and good practice to order medication at standard regular intervals in palliative care contexts, as this is based on half-life of the drug. There are other avenues for responding to pain that is not controlled by regular doses of analgesia e.g. PRN dosing with close monitoring. This is another example of ambiguity of the participants' meaning that requires more explanation. | The nurse was working in the terminal-cancer ward, and residents in rotation were in charge of taking care of the patients, who were not specialists in palliative care, including PRN dosing. The nurse indicated both the lack of palliative care specialist and the lack of palliative care education. We have clarified this point. |
| Discussion: - More specific examples/details of similar/different findings from other studies is required, as the only one mentioned was about job retention. | We have added more details in this section to make comparisons. |
| - Second paragraph, page 16: here again the focus is attempting to be too broad. What is unique and interesting about your study is that it was set in in South Korea and has specific implications for the health care system there. Please revise this section so that it becomes more appropriately focused on the context within which the study was conducted. | Thank you for your suggestion. We have deleted the paragraphs. |
| - Please state limitations of your study at the end of the discussion. | Thank you for your suggestion. We have added the limitations of this study. |
| Alignment with COREQ: Items 4,5,6,7,8,25, 28,29 and 30 have not been adequately addressed. I strongly encourage that you do so to ensure your study reporting is of the highest quality. | Thank you for your suggestion. We have added the items within the revised manuscript. However, we could not find a good place to insert some items and would like to include them here:  No. 7: Participants came to know about the interviewer’s name, occupation, reasons for doing this research, and marital status.  No 8: The interviewer was a medical anthropology doctoral candidate and this character contributed to asking participants from outsider perspective.  No 25: The authors did not provide a description of the coding tree.  No 28: Participants did not provide feedback on the findings.  No 30: There was consistency between the data presented and the findings. |
| Reference 1 has a punctuation error. | We are sorry to miss this. We have made sure all references right. |
