# Supplementary material for: Care for critically and terminally ill patients and moral distress of physicians and nurses in tertiary hospitals in South Korea: A qualitative study
Source: PLoS One. 2021 Dec 16;16(12):e0260343. doi: 10.1371/journal.pone.0260343 (PMC8675648; doi:10.1371/journal.pone.0260343)
Supplement: Attachment — Submitted filename: Response to Reviewers.docx [file pone.0260343.s003.docx]

**Response to Reviewers**

**Reviewer #2**

| **Comments** | **Response** |
| --- | --- |
| I congratulate the authors on a very well written and deeply moving account of the findings of their qualitative study, and wish them well in your ongoing work in this important area of patient care. | We appreciate your congratulations. It has been a year-long round of review. Your comments truly helped us to improve the manuscript. We are very glad that you think this manuscript is finally ready to go. We wish we can find a good opportunity to work with you some day. |

**Reviewer #4**

| **Comments** | **Response** |
| --- | --- |
| The article has addressed the outstanding concerns but would benefit from a structured abstract with a more considered conclusion. | Thank you very much for your suggestion. We have revised the abstract so that we can more concisely convey the points we addressed. |

**Reviewer #5**

| **Comments** | **Response** |
| --- | --- |
| In this paper, a qualitative study describes the ethical conflicts of medical staff in Korean acute care hospitals. Qualitative research has been conducted by taking over the appropriate method, but I have some problems about the interpretation of the results. 1. The results of the interview are the five things shown in Table 3, but the conclusion is that 'As a result, the participants went through stress, lack of enthusiasm, guilt, depression, and skepticism.' are written and there is a leap in interpretation. | Thank you for your suggestion. In the previous version, we had included a section in the end of the Results chapter, the title of which is “Outcomes of moral distress.” This section addresses what the participants went through. We have also added Table 4 in the “Outcomes of moral distress” section to clearly convey this point. |
| 2. This study reveals much the same results as similar previous studies, as the authors have stated in their discussions. Therefore, I cannot find the strength or novelty of this research. I suggest that the author examine the results more deeply and consider the categorized list. | Thank you for your feedback. It is true that many parts of the results of this study are consistent with previous studies, but we do not believe that this means that our study lacks any strength or novelty. As noted, studies concerning moral distress involving in end-of-life care have mainly been conducted in Euro-American areas and in nursing homes/hospices. This study contributes to the discourse by adding data collected in East Asia. The fact that this study found many similarities to previous studies may indicate that end-of-life care causes moral distress in any context. Furthermore, this may be attributed to the feature of the hospital system in our era rather than “cultural differences.”  In addition, this study addresses “saving lives” and “lack of comfort care after stopping life-sustaining treatment,” which rarely appear in previous studies. This indicates that large-scale acute care settings generate some different kinds of moral distress compared to nursing homes and hospices.  Following your advice, (1) we have added Table 4 in the “Outcomes of moral distress” section; (2) and have revised Discussion and Conclusion chapters so that the implications of this study are highlighted. We appreciate your comments and hope that this statement and the revision are sufficiently persuasive. |
